# Quantitative T1 mapping of the normal brain from early infancy to adulthood

**DOI:** 10.1007/s00247-020-04842-7

**Published:** 2020-10-17

**Authors:** Daniel Gräfe, Jens Frahm, Andreas Merkenschlager, Dirk Voit, Franz Wolfgang Hirsch

**Affiliations:** 1 Department of Pediatric Radiology, University of Leipzig, Liebigstraße 20a, 04103 Leipzig, Germany; 2 Biomedizinische NMR, Max-Planck-Institut für biophysikalische Chemie, Göttingen, Germany; 3 Department of Pediatrics, University of Leipzig, Leipzig, Germany

**Keywords:** Adolescents, Brain, Children, Magnet resonance imaging, Normal values, T1 mapping

## Abstract

**Background:**

Quantitative mapping of MRI relaxation times is expected to uncover pathological processes in the brain more subtly than standard MRI techniques with weighted contrasts. So far, however, most mapping techniques suffer from a long measuring time, low spatial resolution or even sensitivity to magnetic field inhomogeneity.

**Objective:**

To obtain T1 relaxation times of the normal brain from early infancy to adulthood using a novel technique for fast and accurate T1 mapping at high spatial resolution.

**Materials and methods:**

We performed whole-brain T1 mapping within less than 3 min in 100 patients between 2 months and 18 years of age with normal brain at a field strength of 3 T. We analyzed T1 relaxation times in several gray-matter nuclei and white matter. Subsequently, we derived regression equations for mean value and confidence interval.

**Results:**

T1 relaxation times of the pediatric brain rapidly decrease in all regions within the first 3 years of age, followed by a significantly weaker decrease until adulthood. These characteristics are more pronounced in white matter than in deep gray matter.

**Conclusion:**

Regardless of age, quantitative T1 mapping of the pediatric brain is feasible in clinical practice. Normal age-dependent values should contribute to improved discrimination of subtle intracerebral alterations.

**Electronic supplementary material:**

The online version of this article (10.1007/s00247-020-04842-7) contains supplementary material, which is available to authorized users.

## Introduction

In diagnostic imaging, quantitative parametric mapping is a prerequisite for any objective tissue characterization. In MRI this mainly refers to a determination of the true relaxation times T1, T2 or T2*. In contrast to common MRI sequences, where the measured signal intensity reflects a weighted mixture of the spin density with T1, T2 and T2* contrast, the “intensity” of a parametric map represents a biochemically defined tissue characteristic in an anatomically defined location. For example, the absolute T1 relaxation time within different anatomical structures of the child’s brain depends on the local microstructural environment and tissue composition. It therefore provides access to a range of cellular properties including fluid content, degree of myelination, cellular density, phospholipids, proteins, fat and paramagnetic substances such as iron or contrast agents [1–3].

Early expectations that MRI relaxation times might lead to an equivalent for the Hounsfield units in X-ray CT have not been fulfilled, for several reasons. First, the experimental gold standard for the assessment of T1 is a spin-echo inversion-recovery sequence, which is not suitable for routine use because of its very long acquisition time. Faster T1 mapping techniques usually employ multi-echo readout modules that are very sensitive to magnetic field inhomogeneity. Moreover, even at the same field strength, T1 dependencies on sequence type or manufacturer hint to systematic deviations from the true T1 value [4]. Other limitations relate to a poor spatial resolution or a lack of multi-slice acquisitions. These problems might also contribute to observations that reported T1 values of healthy tissues overlap with pathologies such as inflammation and edema [5]. On the other hand, and despite these limitations, a diagnostic benefit of quantitative T1 mapping of the brain has been convincingly demonstrated for a number of conditions, including hepatic encephalopathy [6], multiple sclerosis [7–9], sickle cell anemia [10], epilepsy [11], heavy metal deposition [12], neurofibromatosis [13], brain tumor follow-up [14, 15], infantile brain development [16–18] and aging [19]. The continuing desire for a viable quantitative MR sequence is also reflected in the multitude of recent faster mapping approaches, such as the MP2RAGE sequence [20], synthetic MRI [21] and MR fingerprinting [22].

Here we applied a novel approach to very fast and accurate T1 mapping at high spatial resolution that promises to overcome many of the limitations mentioned. The technique is based on a single-shot inversion-recovery sequence with only a single 4-s real-time MRI readout that comprises a series of images describing the inversion-recovery process [23]. This portion employs a highly subsampled radial fast low-angle shot (FLASH) sequence with image reconstruction by regularized nonlinear inversion (NLINV) as originally developed for dynamic real-time MRI [24]. T1 maps are then calculated by pixelwise fitting. The use of a slice-selective inversion pulse allows for sequential multi-slice capabilities so that, depending on the chosen slice thickness and covered volume, T1 maps of the entire brain without gaps can be obtained in less than 3 min. Both accuracy and precision have been validated using numerical and experimental phantoms [23, 25]. It is technically supported by the use of a real-time FLASH readout with random radiofrequency spoiling [26], which avoids inconsistent steady states of steady-state free precession (SSFP)-based variants as well as any sensitivity to magnetic field inhomogeneity because of the use of a single gradient echo with the shortest possible echo time. The method for T1 mapping works at 3 tesla (T) and 1.5 T. The employed technique can be integrated into different MRI systems, although the current implementation is only available for Siemens MRI systems (Siemens Healthcare, Erlangen, Germany).

Our aim was to determine normal T1 values at 3 T for different brain regions of gray and white matter in infants, children and adolescents using this improved T1 mapping technique. These values can serve as a basis for the characterization and differentiation of myelination disorders and other pathologies of the dynamically developing brain during the first 18 years of life.

## Materials and methods

Our local ethics committee approved this study. Patients or parents signed informed consent regarding publishing their data and photographs.

### Patient cohort

We included 100 patients ages 2 months to 18 years who received an MRI exam with T1 mapping at our hospital between October 2019 and March 2020. Inclusion criteria required conventional MRI sequences (T1, T2, T2 fluid-attenuated inversion recovery, diffusion-weighted imaging, and if necessary T1 after administration of intravenous gadolinium) to be classified as normal by consensus of two experienced pediatric radiologists (F.W.H. and D.G., with 25 years and 8 years of MRI experience, respectively) (Table 1). Exclusion criteria were a history of gadolinium administration, more than mild neurologic symptoms that had led to the MRI/T1 mapping indication such as developmental regression or pathological electroencephalogram, history of cerebral tumors or proven brain-related diseases such as metabolic disorders or neurofibromatosis. We excluded 318 T1 mapping studies of patients with such medical history or with MR-detectable pathology. We verified an age-appropriate development of the white and gray matter by comparing with a neurologic atlas [27].

**Table 1:** Characteristics of patients included for T1 mapping, all of whom had normal findings in conventional cranial MRI

Indication	*n*	Male:female	Age in years, mean (range)
Primary headache	39	17:22	11.8 (1.1–18.0)
Suspected seizure	13	5:8	8.3 (1.5–16.1)
Neurologic abnormalities^a^	17	11:6	8.4 (0.2–17.6)
Psychiatric abnormalities	8	2:6	13.9 (10.1–17.6)
Trauma	4	2:2	12.0 (5.6–17.2)
Skull anomaly	4	2:2	0.7 (0.5–0.9)
Developmental delay without regression	3	1:2	5.8 (0.7–8.7)
Other pathologies with unremarkable neurocranium^b^	12	6:6	8.6 (0.5–15.3)
**Total**	**100**	**46:54**	**9.8 (0.2–18.0)**

^a^Among others: nystagmus, gait disorder, ataxia, tics, dizziness

^b^Among others: cleft palate, spina bifida, immune deficiency, orbital vascular malformations, uveitis, cerebral vascular variants

### Image acquisition

All examinations were performed on a 3-T MRI system (Prisma^fit^; Siemens Healthcare) with a 64-channel head coil. Basic technical details of T1 mapping have been described elsewhere [23]. Extending the original work, the present variant acquires a single-slice with use of a single-shot inversion recovery sequence that comprises a slice-selective inversion pulse followed by a 4-s FLASH MRI readout yielding a series of high-speed images. These highly undersampled radial acquisitions (repetition time/echo time [TR/TE] = 4.0/2.2 ms) with a small golden-angle trajectory (20.9°) employ a flip angle of 6° as well as randomized radiofrequency spoiling [26]. The use of 15 radial spokes per image leads to a temporal footprint of 60 ms for sampling the inversion-recovery process (i.e. corresponding to 67 images for a 4-s scan). The slice thickness was 3 mm with an in-plane resolution of 1×1 mm covering a 220×220-mm^2^ field-of-view in transverse orientation. Usually 40 sections were acquired within a total measuring time of 2 min 50 s. The nonlinear inverse image reconstruction was performed online on a graphics processing unit cluster with eight graphical processing units (total reconstruction time for all sections about 4 min).

### Image analysis

**Fig. 1:**
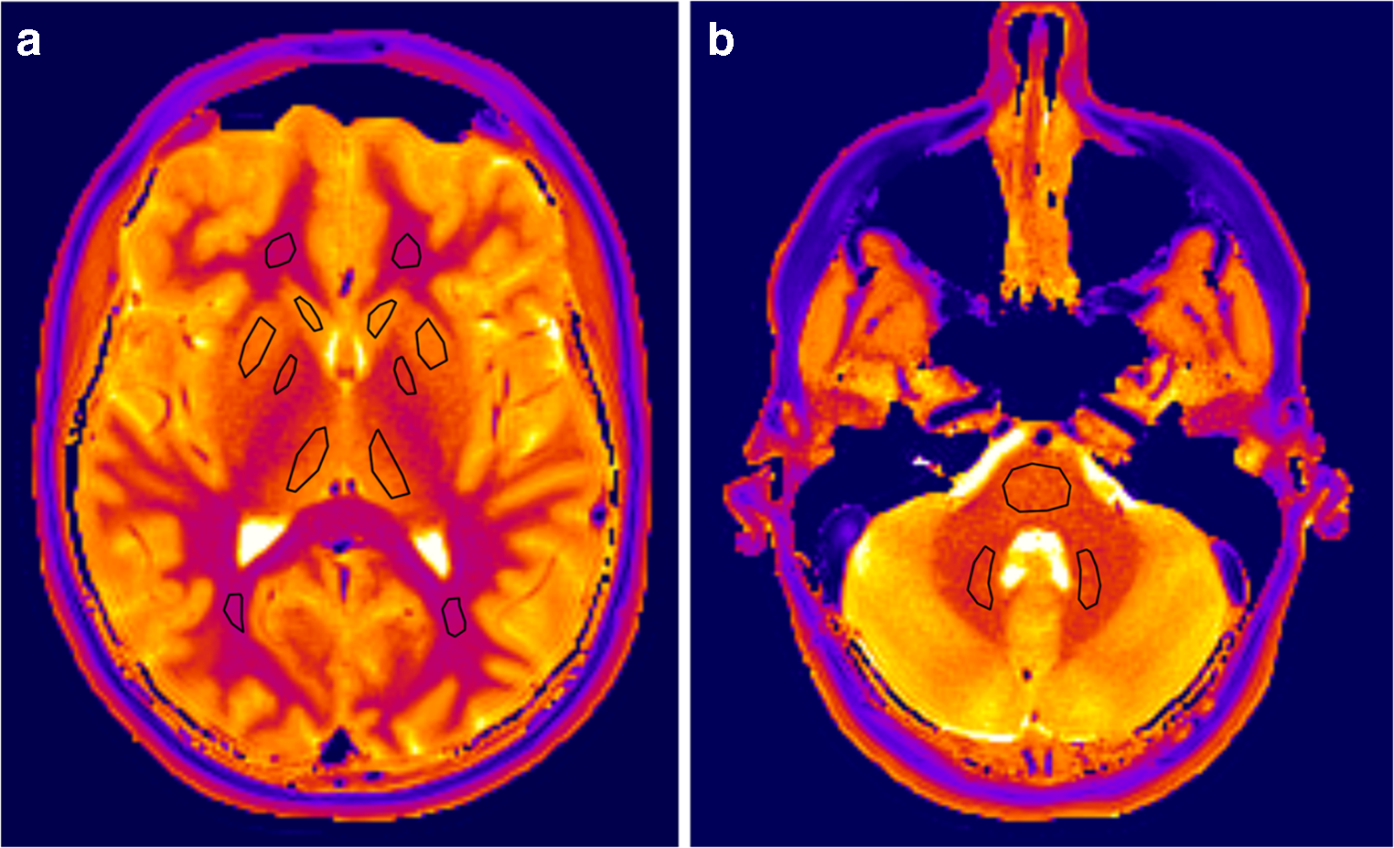
Representative axial T1 maps of the brain of a 9-year-old boy at the level of (**a**) the basal ganglia and (**b**) the fourth ventricle with definition of regions-of-interest

Two observers independently determined regional T1 relaxation times by manually drawing the largest possible region-of-interest (ROI) for a particular structure using conventional radiologic image processing (IntelliSpace Portal; Philips, Best, the Netherlands). They evaluated the following locations: nucleus caudatus, putamen, globus pallidus, thalamus, nucleus dentatus, frontal white matter, occipital white matter, brainstem at the level of the fourth ventricle just below the pons and the bulbus oculi (Fig. 1). To determine the intra-rater variability, the observers assessed the same region in the left and right hemisphere; no differences were expected according to previous studies [16, 28]. The mean and standard deviation of the T1 values per ROI were determined.

### Statistics

We compared different regression models (linear, logarithmic, exponential, square root) and calculated the adjusted coefficient of determination R^2^. After linear regression over the transformed data, we investigated normal distribution of the residuals in the quantile plot. Subsequently, we determined the 2.5th and 97.5th percentiles using the table of standard normal distribution as confidence interval. The intra-rater variability (homologue brain areas) and the inter-rater variability (averaged values of both raters) were examined by a Bland–Altman analysis. Additionally, we calculated the interclass coefficient. The significance of T1 differences between structures was quantified by a Wilcoxon rank sum test. The calculations and graphical plots were performed with software R (R Foundation for Statistical Computing, Vienna, Austria) and RStudio (RStudio Inc., Boston, MA).

## Results

**Fig. 2:**
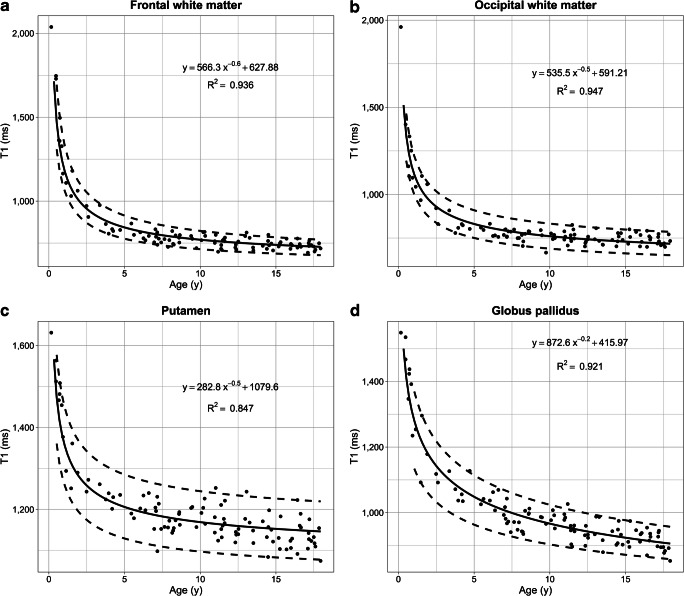

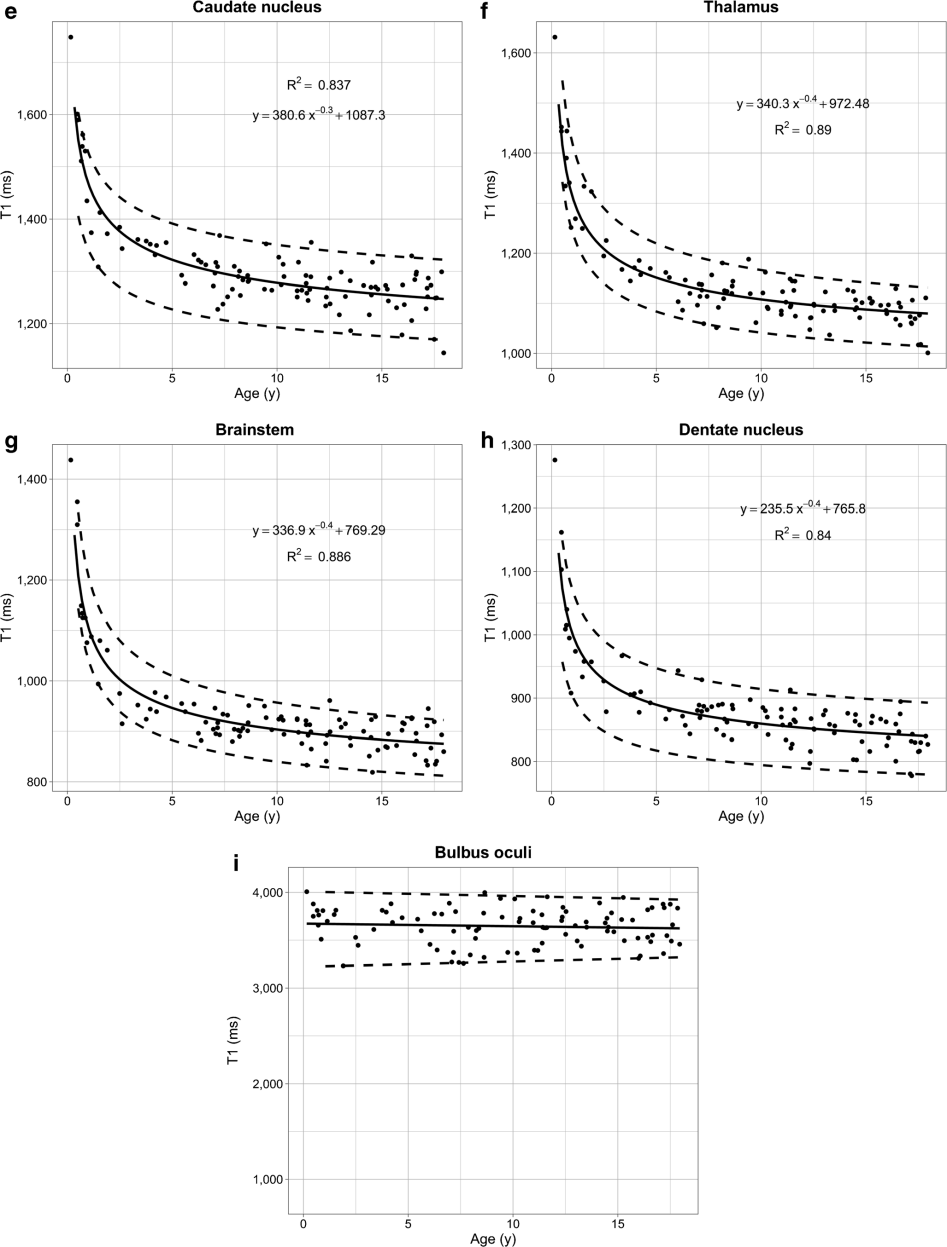
T1 relaxation times in different brain regions of children with inconspicuous cranial MRI as a function of age. **a** Frontal white matter. **b** Occipital white matter. **c** Putamen. **d** Globus pallidus. **e** Caudate nucleus. **f** Thalamus. **g** Brainstem. **h** Dentate nucleus. All regions reveal a steep decrease of T1 values with age, with an exponential characteristic. **i** For comparison, the age-independent water proton T1 relaxation times of bulbus oculi is also demonstrated. *Solid lines* represent the regression function of the mean. *Dashed lines* indicate the 95% confidence interval

The T1 relaxation times of the pediatric brain rapidly decrease in all regions within the first 3 years of age, followed by a significantly slower reduction until adulthood (Fig. 2). This age-dependent course of T1 values can be described by an exponentiation function ax^r^ + b with fractional (negative) power r = −n/10. The steepest decline was observed for the frontal and occipital white matter. Especially in the first 6 months of age, the T1 relaxation times varied substantially. The R^2^ after hyperbolic transformation ranged from 0.84 to 0.94 (*P*<0.001 for each region). Differences in T1 values between individual structures were all highly significant (*P*<0.01). Bland–Altman plots for inter-rater variability are shown in Online Supplementary Resource 1. Both intra-rater reliability and inter-rater variability were in most cases excellent (intraclass correlation coefficient [ICC] > 0.92). The inter-rater reliability in bulbus oculi was good (ICC 0.78).

## Discussion

We present normative values for T1 relaxation times in several representative regions of the cerebral gray and white matter over the entire pediatric age range using a novel method for fast and accurate T1 mapping. The employed technique can be easily integrated into clinical routine because of its short acquisition time. The signal-to-noise ratio of the T1 maps is very high for the parameters chosen here, i.e. a spatial resolution of 1 mm in-plane and a slice thickness of 3 mm. In general, however, further adaptations are possible in order to meet specific clinical demands. Because of the high spatial resolution, even smaller anatomical regions can be mapped precisely, which is reflected in a very good intra-rater and inter-rater reliability.

The wider scattering of T1 values in the bulbus oculi can be explained by contamination by involuntary eye movements in some patients. T1 values of cerebrospinal fluid (CSF) spaces did not qualify as reference because of their broad dispersion. This was most likely caused by partial volume effects because the inner and outer CSF spaces are smaller in children than in adults. Furthermore, in larger CSF spaces T1 values might be affected by residual fluid movements during the 4-s acquisition.

We found a very rapid decrease in T1 relaxation times in each region of the brain within the first 2 years of age, which turned into a slight linear decrease from about 5 years of age until adulthood. The initial drop was most pronounced in white matter and can be explained by its dynamic maturation, especially from myelination. In contrast to the literature, our data suggest that the initial steep and late flat drop can be described more closely by a reciprocal exponential function than by a logarithmic function. Deoni et al. [16] analyzed the degree of myelination, more precisely the myelin water fraction, in the white matter of 153 infants and toddlers ages 3–60 months. Their T1 determinations relied on the mcDESPOT technique, which represents FLASH acquisitions with variable flip angles, which are known to have inconsistent slice profiles. Moreover, a comparison with the present data is precluded because no direct T1 values have been reported. Eminian et al. [18] measured T1 relaxation times of gray and white matter in 42 children ages 1–20 years using MP2RAGE. Four regions matched those in our study: the nucleus caudatus, putamen, thalamus and frontal white matter. Compared to our results, the T1 relaxation times recorded by Eminian seem similar but slightly higher on average (pooled data of children of 5 years and older: putamen 1,172 ms versus 1,162 ms, nucleus caudatus 1,297 versus 1,272 ms, thalamus 1,081 ms versus 1,101 ms, frontal white matter 806 versus 758 ms and more dispersed. Recently Lee et al. [29] presented normal values for T1 and T2 relaxation as well as proton density in a pediatric cohort of 89 children with a median age of 18 months with a synthetic MRI sequence. Concordant to our results, this study again demonstrated the very rapid drop in T1 relaxation time in the first years followed by a much slower decline until adulthood. Between Lee’s and our study, three regions of interest overlapped. The authors split their cohort into different age groups. The T1 relaxation times in the age group with the least decrease (i.e. older than 5 years) were on average slightly lower than the results in our study: in the frontal white matter 707 ms versus 758 ms, in the thalamus 951 ms versus 1,101 ms, and in the nucleus caudatus 1,160 versus 1,272 ms. While deviations for older children probably reflect systematic technical differences as previously suggested [4], differences in the lower age range might additionally be caused by the comparatively low number of cases. In our work, separate evaluations for boys and girls were omitted because of the expected minimal differences and the significantly higher number of cases otherwise needed.

The T1 mapping technique used here turns out to be a highly promising tool for integrating pediatric T1 mapping into routine clinical protocols. It will contribute to objectifying and quantifying dynamic developmental processes in the child’s brain and related pathologies. Moreover, apart from applications to the brain, single-shot T1 mapping can also be applied to the heart, where diastolic T1 maps can be obtained with use of pulse wave triggering and automatic deletion of systolic frames. Further extensions apply to the characterization of all pathological processes in parenchymatous organs. Because of the remarkably fast acquisition time and the high reliability of the T1 values, the sequence promises applications with low threshold in everyday practice.

Correlations of T1 relaxation times with voxel-based methods or tract-based methods in a specialized research setting might provide valuable data for understanding pathogenesis or cerebral maturation processes [3]. However, because of their technical complexity, these methods have not been suitable for routine use. In contrast, the easy and direct evaluation of regional T1 values from quantitative maps offers the benefit that no dedicated software and no time-consuming data preparation are required. In diagnostic imaging, multi-parametric quantitative MRI is gaining more and more attention, driven also by the progress in machine learning. For this purpose, a maximum amount of normal and pathological relaxation data is desired, so that a fast, simple and safe technique for acquiring and evaluating cerebral T1 relaxation times is a promising strategy.

## Conclusion

We demonstrated the application of a new T1 mapping technique for the brain of children and established age-dependent normal values. This method, derived from real-time MRI, makes it possible to generate accurate high-resolution T1 maps of the pediatric brain in only a fraction of the time required with conventional T1 mapping sequences. The collected age-dependent T1 relaxation times for normal brain will serve as a basis for further quantitative, possibly also multi-parametric, MRI diagnostics of the brain in children.

## Electronic supplementary material

Online Resource 1 Bland–Altman plots for inter-rater variability in different brain regions (**a–i**) (PDF 5 kb)

(PDF 5 kb)

(PDF 5 kb)

(PDF 5 kb)

(PDF 5 kb)

(PDF 5 kb)

(PDF 5 kb)

(PDF 5 kb)

(PDF 5 kb)
