# Supplementary figures and images for: Quantitative T1 mapping of the normal brain from early infancy to adulthood
Source: Pediatr Radiol. 2020 Oct 17;51(3):450–6. doi: 10.1007/s00247-020-04842-7 (PMC7897197; doi:10.1007/s00247-020-04842-7)

# Frontal white matter

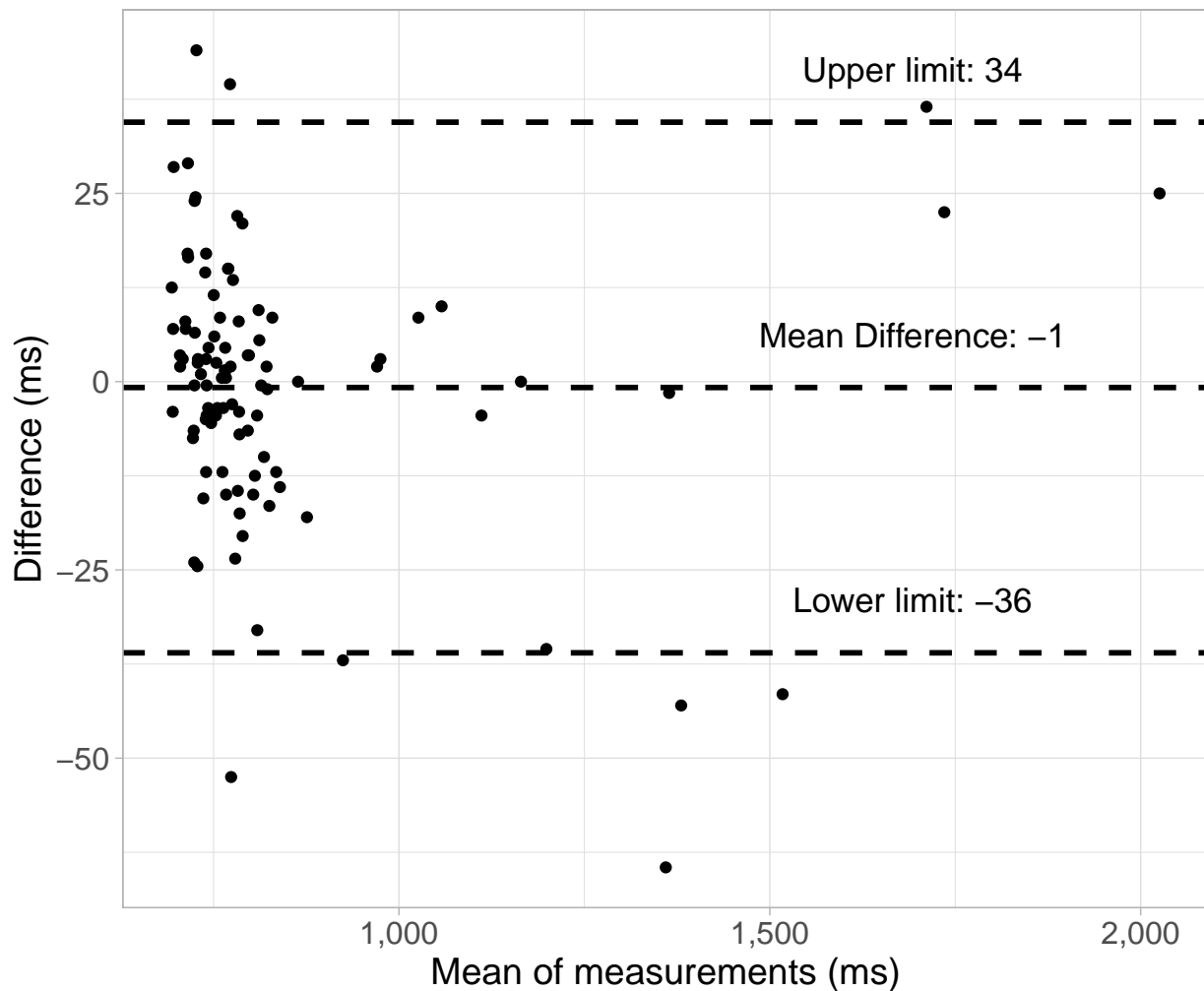

Supplement: Supplementary file 1 — Bland–Altman plots for inter-rater variability in different brain regions (a–i) (PDF 5 kb) [file 247_2020_4842_MOESM1_ESM.pdf]

# Occipital white matter

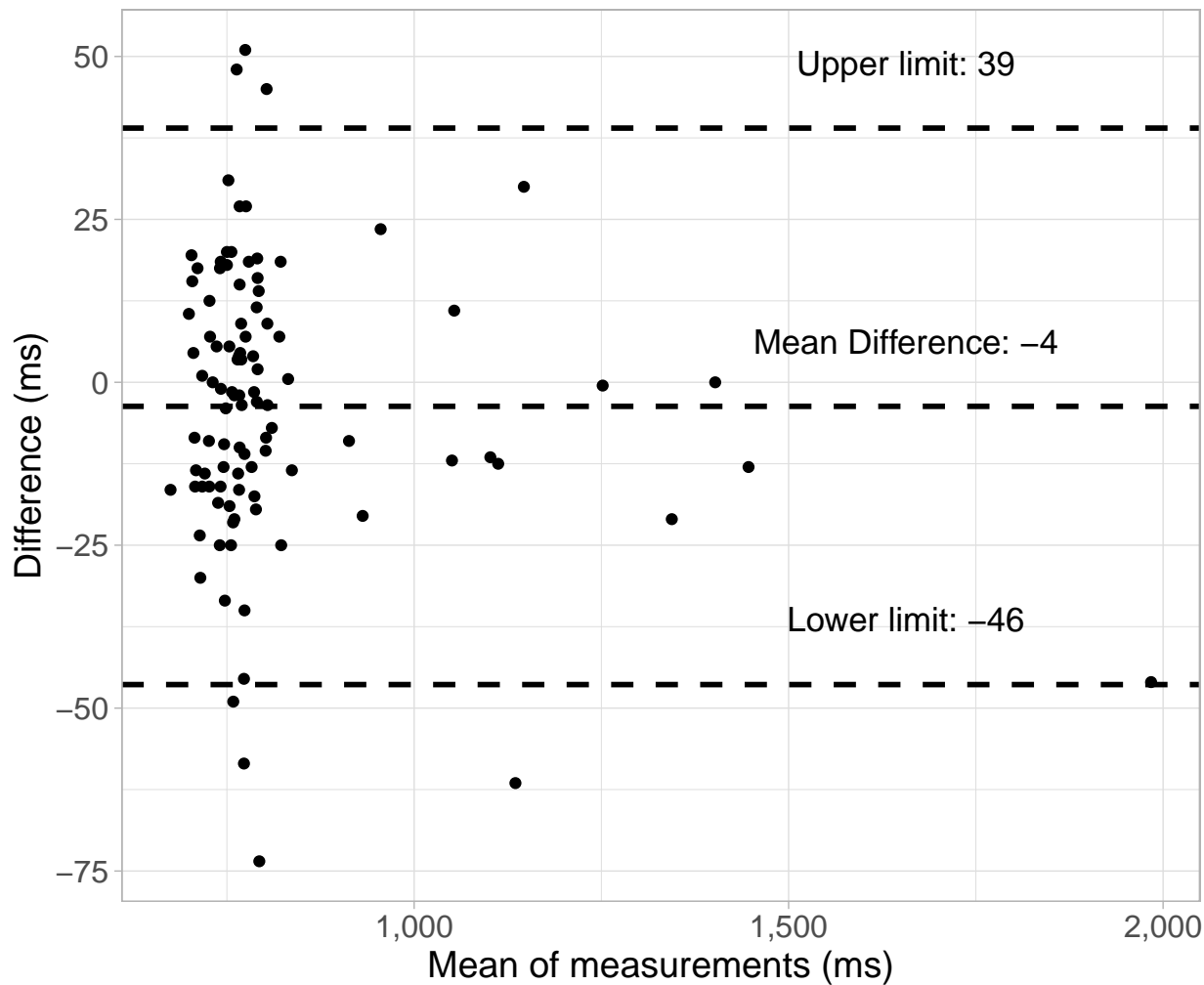

Supplement: Supplementary file 2 — (PDF 5 kb) [file 247_2020_4842_MOESM2_ESM.pdf]

# Putamen

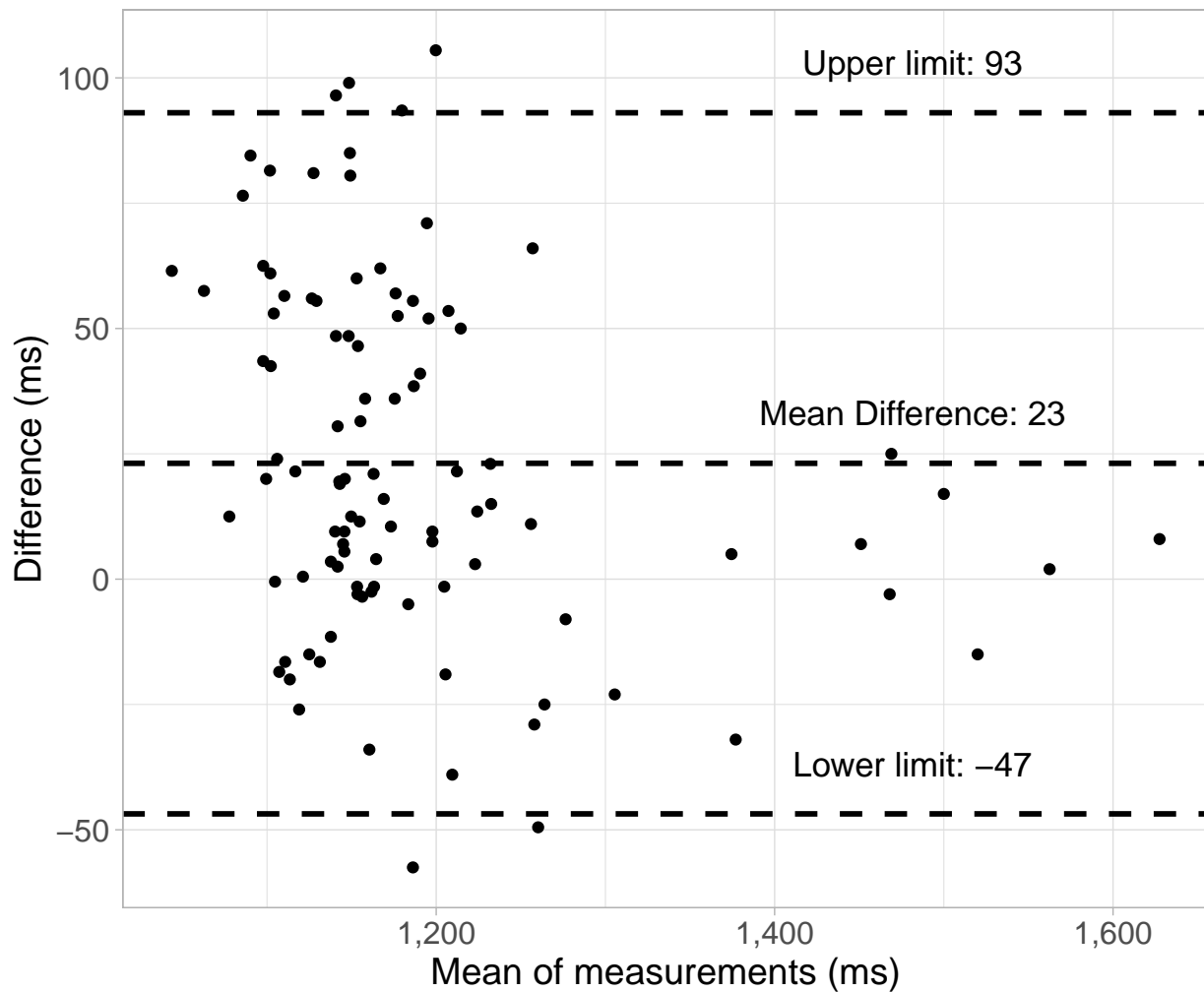

Supplement: Supplementary file 3 — (PDF 5 kb) [file 247_2020_4842_MOESM3_ESM.pdf]

# Globus pallidus

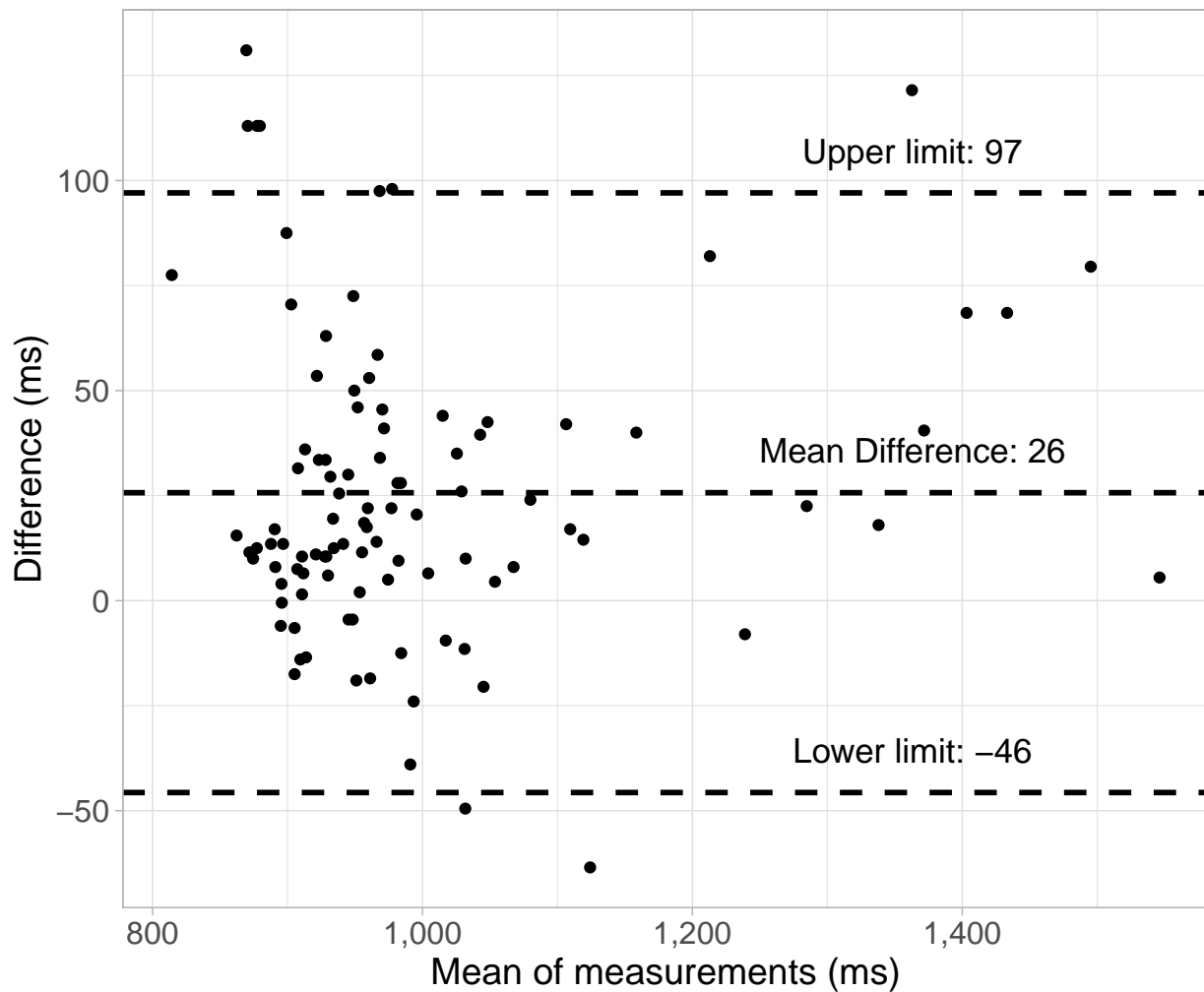

Supplement: Supplementary file 4 — (PDF 5 kb) [file 247_2020_4842_MOESM4_ESM.pdf]

# Nucleus caudatus

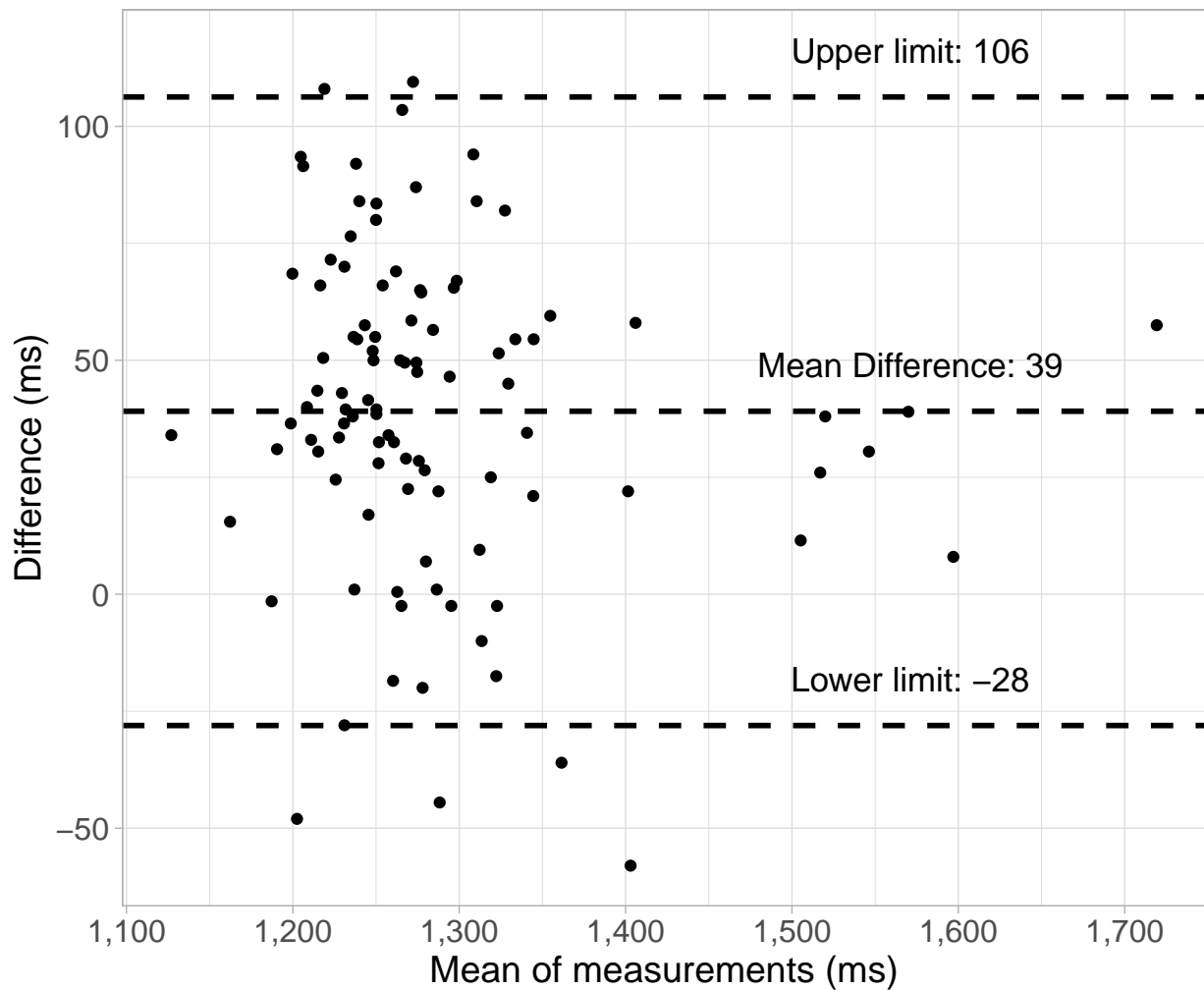

Supplement: Supplementary file 5 — (PDF 5 kb) [file 247_2020_4842_MOESM5_ESM.pdf]

# Thalamus

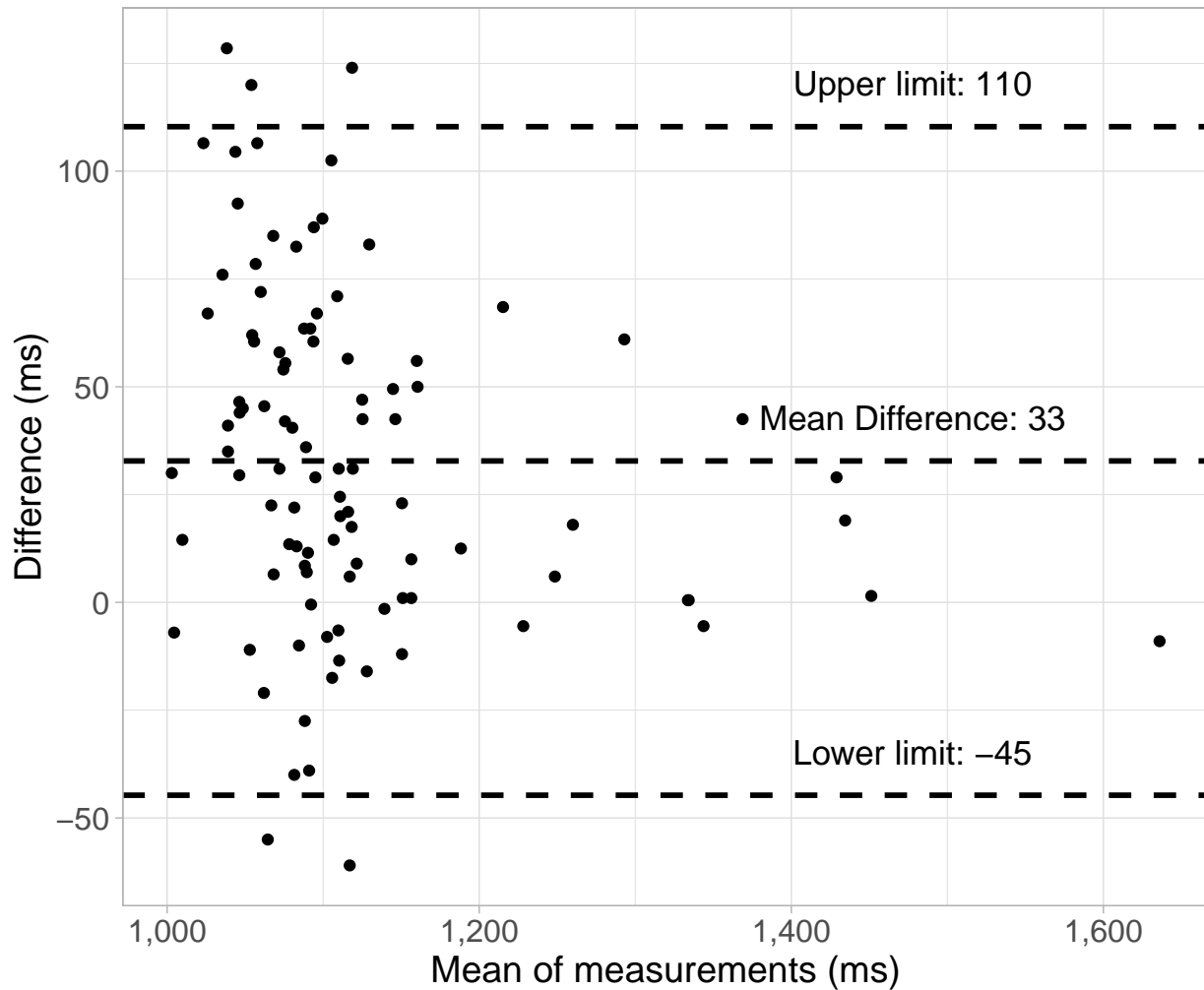

Supplement: Supplementary file 6 — (PDF 5 kb) [file 247_2020_4842_MOESM6_ESM.pdf]

# Stem

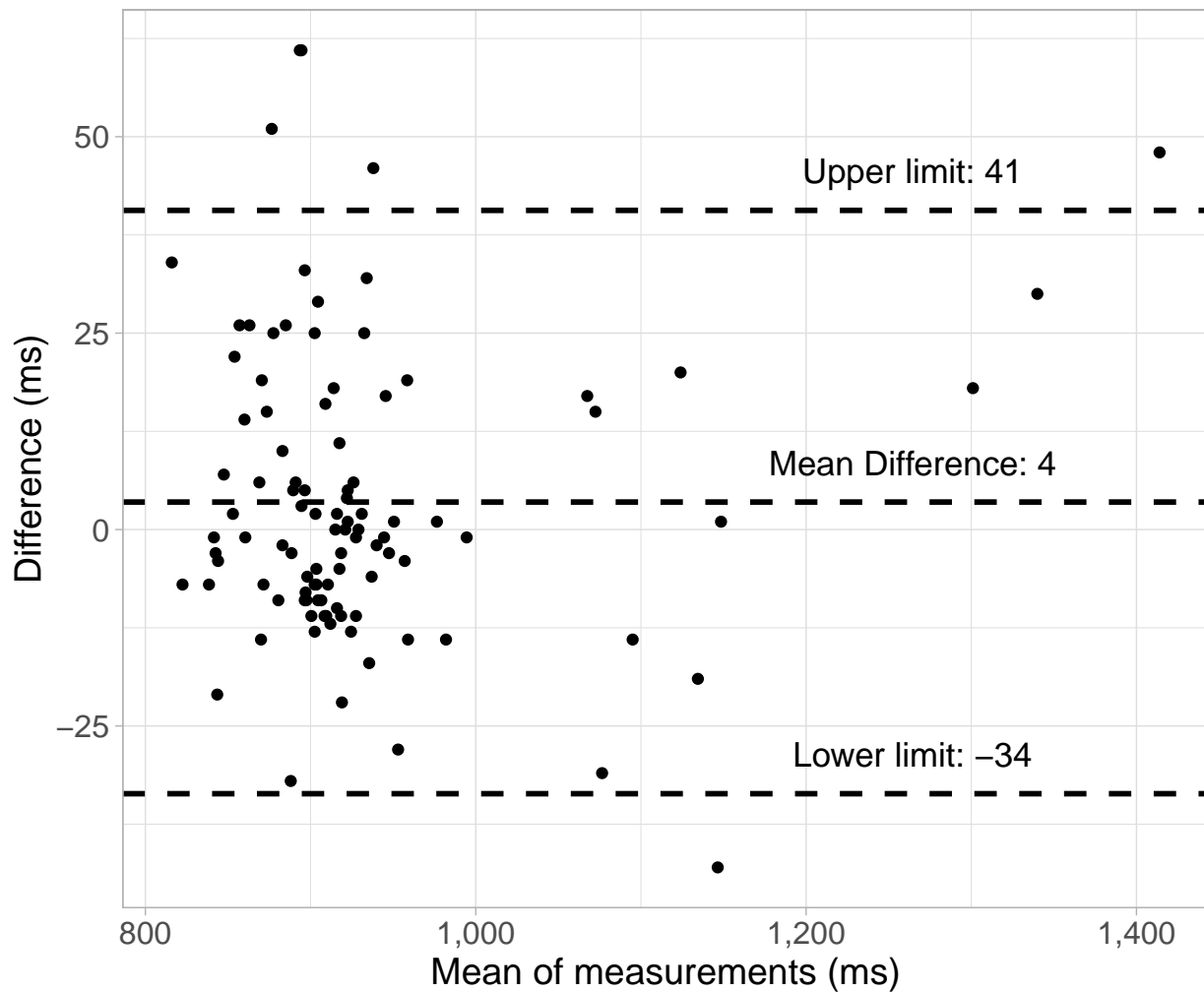

Supplement: Supplementary file 7 — (PDF 5 kb) [file 247_2020_4842_MOESM7_ESM.pdf]

# Dentate nucleus

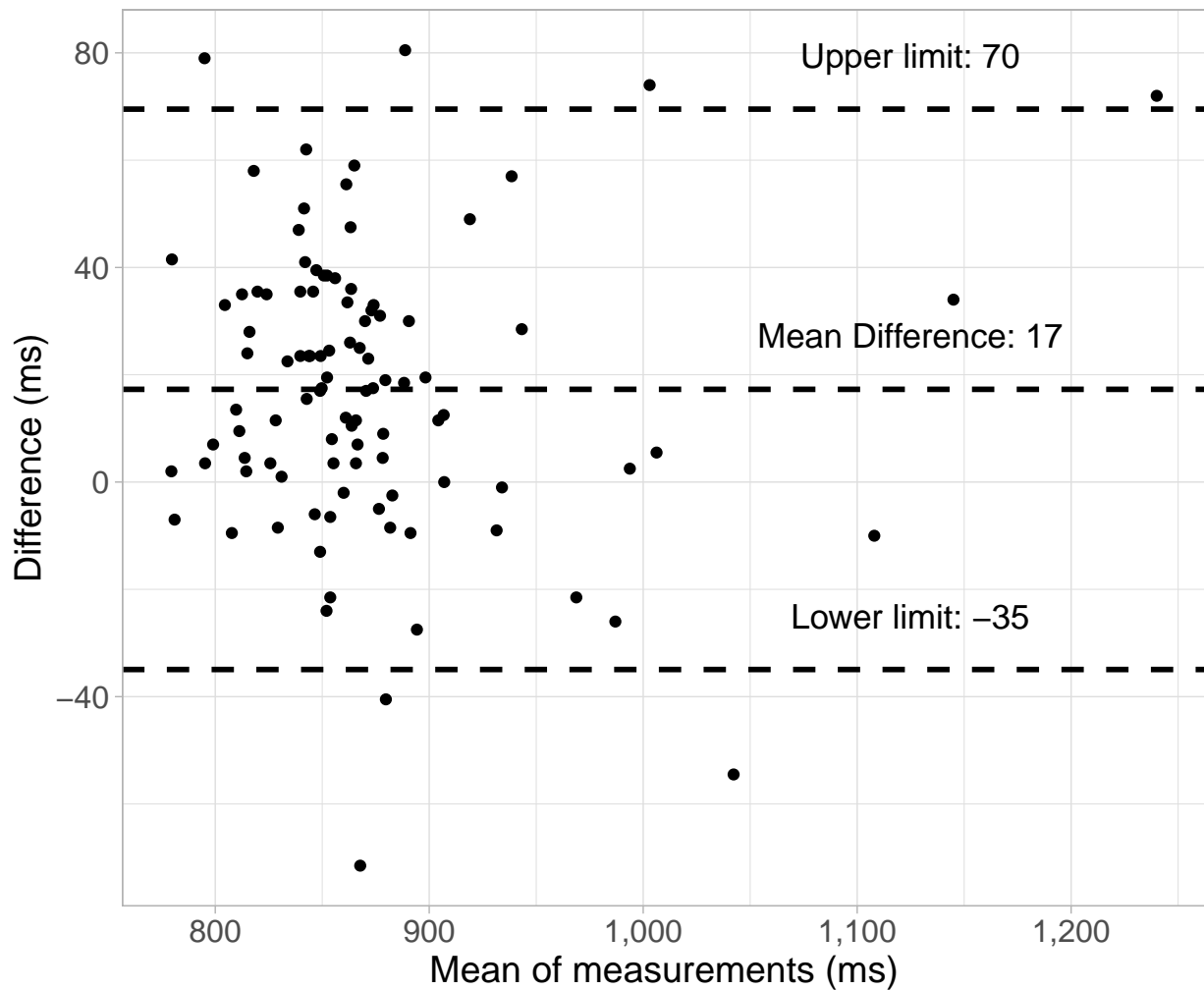

Supplement: Supplementary file 8 — (PDF 5 kb) [file 247_2020_4842_MOESM8_ESM.pdf]

# Bulbus opticus

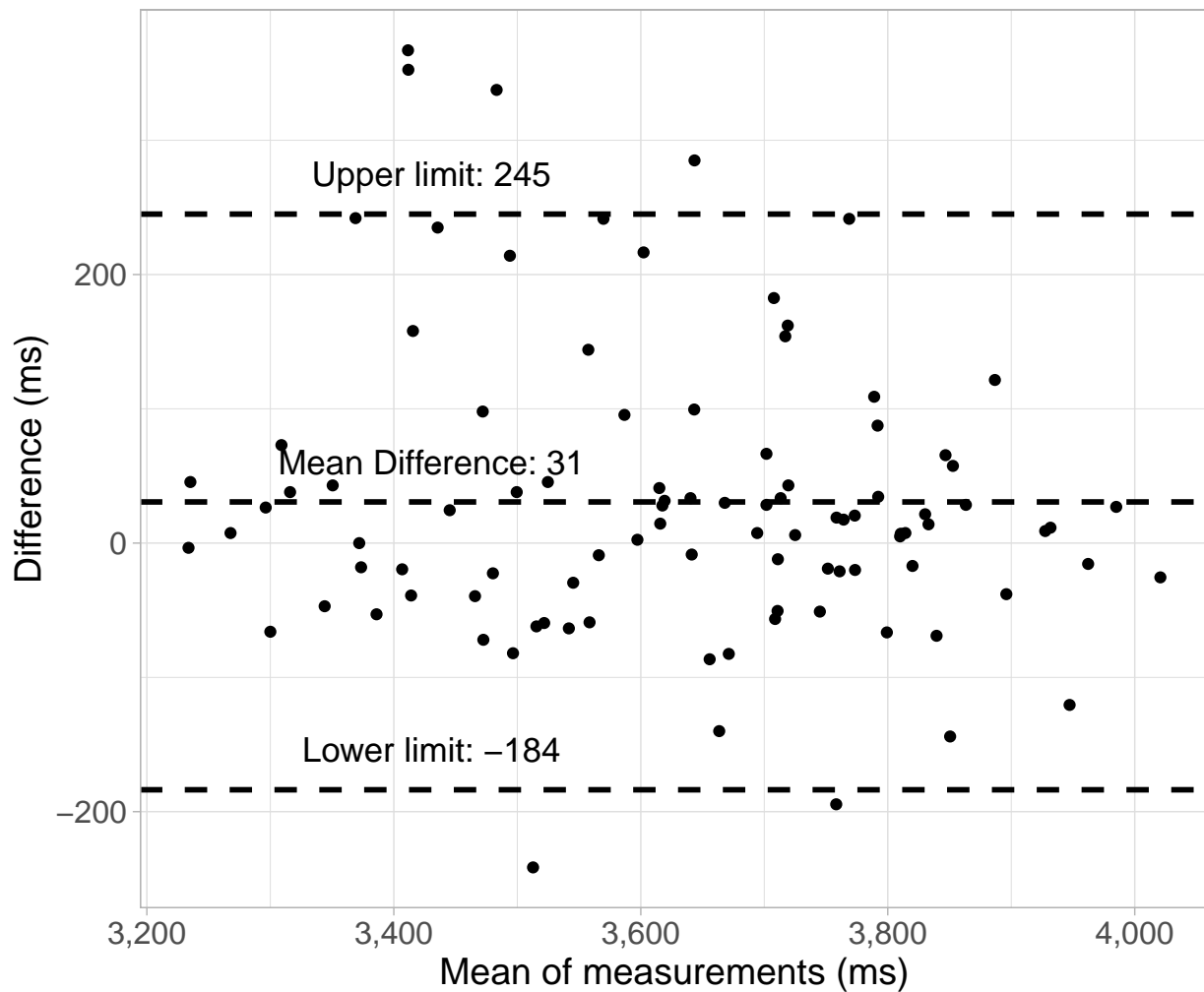

Supplement: Supplementary file 9 — (PDF 5 kb) [file 247_2020_4842_MOESM9_ESM.pdf]
